# Association of intrinsic pathways with altered tumor immune infiltration in hepatocellular carcinoma: New targets for combining immune therapy

**DOI:** 10.1002/ctm2.219

**Published:** 2020-11-16

**Authors:** Zheng Chen, Mincheng Yu, Jiuliang Yan, Binghai Zhou, Wentao Zhang, Lei Guo, Bo Zhang, Shuang Liu, Lei Jin, Jian Zhou, Jia Fan, Qinghai Ye, Hui Li, Yongsheng Xiao, Yongfeng Xu

**Affiliations:** ^1^ Liver Cancer Institute Zhongshan Hospital Fudan University and Key Laboratory of Carcinogenesis and Cancer Invasion Ministry of Education Shanghai People's Republic of China; ^2^ Department of Hepatobiliary and Pancreatic Surgery the Second Affiliated Hospital of Nanchang University Nanchang People's Republic of China; ^3^ Neurosurgery Department of Zhongshan Hospital Fudan University Shanghai People's Republic of China

Dear Editor,

Though cancer immune therapy has achieved benefits in partial patients’ population, the overall responses remain low, and low immune infiltration status has been deemed as a key factor leading to low response.^1^ In hepatocellular carcinoma (HCC), we found that tumor intrinsic oncogenic pathways were related to tumor infiltrating immune cells: mutation of *catenin beta 1* *(CTNNB1)* or activation of the canonical *b‐catenin* pathway was related to poor immune infiltration, while high expression of *Kirsten rat sarcoma viral oncogene homolog (KRAS)* was related to a better immune infiltration status, which provides new thoughts on combining immune therapy by targeting tumor intrinsic pathways and gives new markers for preference of treating methods.

In this study, we studied changed oncogenic pathways, as well as immune infiltration status of tumors, with bioinformatic methods, using publicly available sequencing data and somatic mutation data in TCGA (LIHC) and ICGC (LIRI JP, LICA FR) databases, which covered HCC patients from different ethics and backgrounds (Supplemental Table S1). After calculation of tumor immune infiltration levels of each patient, we found high immune infiltration levels in HCC tissues could predict better survival of HCC patients, and high Th1 cell tumor infiltration was a consistent good indicator for better survival across different datasets (Figure 1). After clustering of patients according to the Th1 cell score, we found in low Th1 cell infiltration patients, genes, related to adaptive immune response signal transduction and T cell stimulation, were suppressed across datasets, and in those patients, signals of the *Wnt/b‐catenin* pathway and *KRAS* downregulated genes were highly enriched (Supplemental Figure S1). After matching sequencing data with somatic mutation information, we found that the mutation status of *CTNNB1* in HCC patients was related to high expression of *CTNNB1* mRNA levels in tumor; in *CTNNB1*‐mutated patients, the *Wnt/b‐catenin* pathway was activated with less infiltrated immune cells in comparison to patients with no *CTNNB1* mutation (Supplemental Figure S2; Figure 2). Besides, in *CTNNB1* mutation patients, genes concerning metabolic changes, such as xenobiotic metabolism, fatty acid metabolism and bile acid metabolism, were also enriched (Supplemental Figure S3).

**FIGURE 1:**
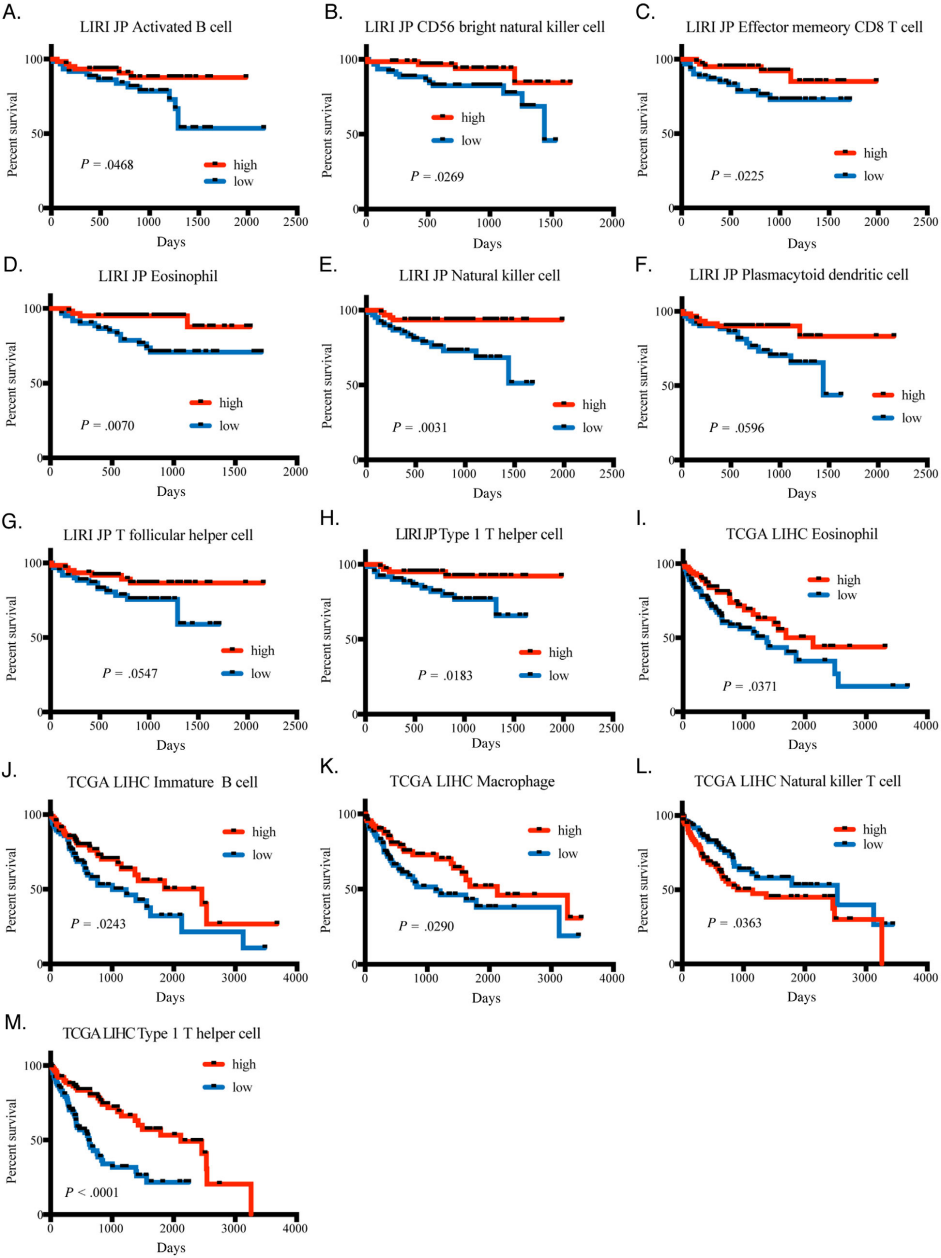
Infiltrating levels of immune cells were correlated to patients’ survival. (A)‐(H) Survival difference between high‐ and low‐activated B cell, CD56 bright natural killer cell, effector CD8+ T cell, eosinophil, natural killer cell, plasmacytoid dendritic cell, T follicular helper cell, and type 1 T helper cell infiltrating groups of LIRI JP project. (I)–(M) Survival difference between high‐ and low‐eosinophil, immature B cell, macrophage, natural killer T cell, and type 1 T helper cell infiltrating groups of TCGA LIHC project

**FIGURE 2:**
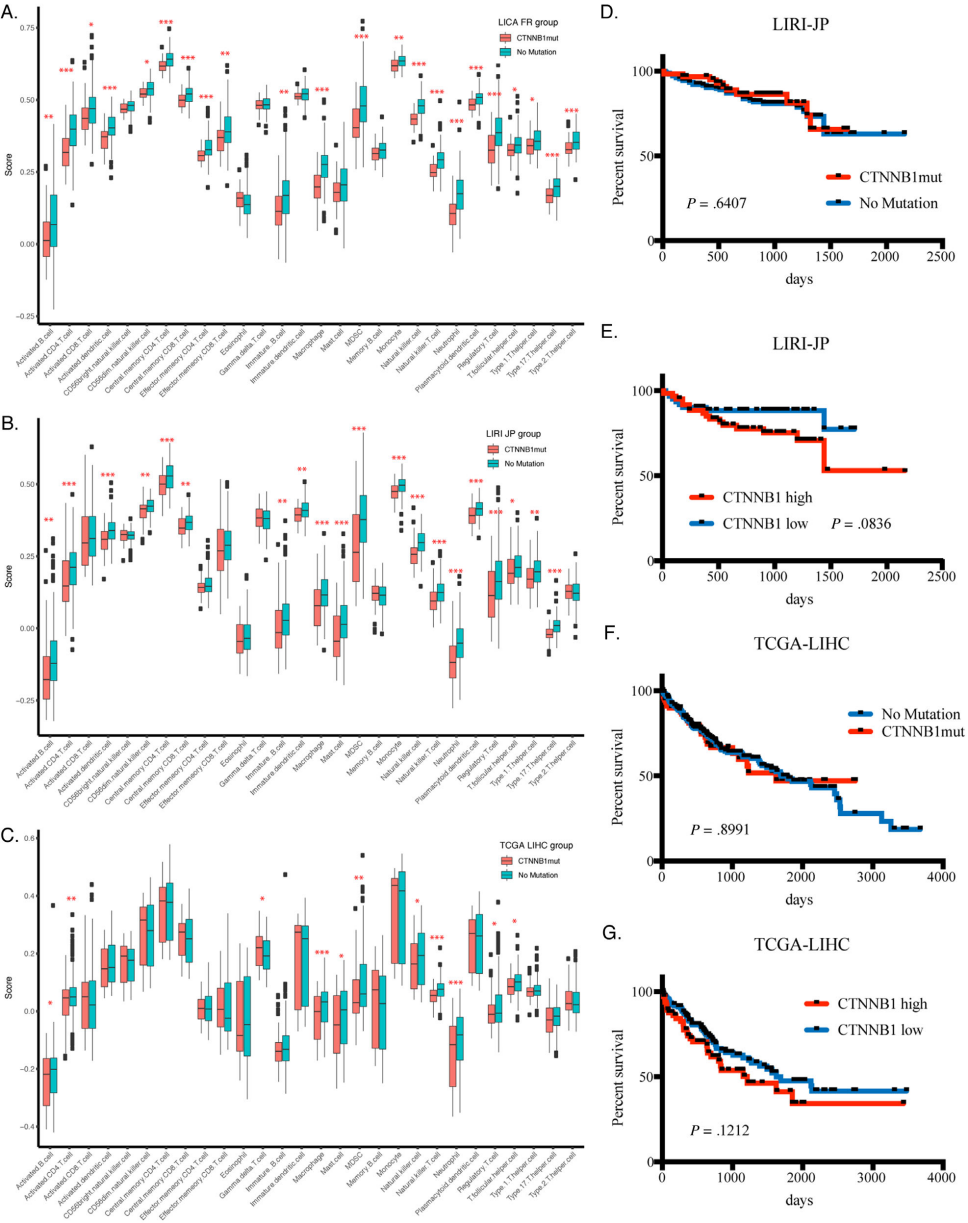
Immune infiltration scores between CTNNB1 mutation and no mutation groups. (A)–(C) Comparison of scores for 28 immune infiltration cell types between CTNNB1 mutation and no mutation groups in projects of LICA FR, LIRI JP, and TCGA LIHC (*p < .05, **p < .01, ***p < .001) (D) Survival curves for CTNNB1 mutation and no mutation groups were similar in project of LIRI JP. (E) Survival curves for high‐ and low‐ CTNNB1 mRNA expression groups showed high CTNNB1 expression group had worse overall survival in project of LIRI JP. (F) Survival curves for CTNNB1 mutation and no mutation groups were similar in project of TCGA LIHC. (G) Survival curves for high‐ and low‐CTNNB1 mRNA expression groups showed high CTNNB1 expression group had worse overall survival in project of TCGA LIHC

Further clustering of patients according to 13 T cell populations showed in the low T cell inflammation group of patients, enriched *KRAS* downregulated genes (including *GPRC5C*, *CPEB3*, *THRB*, *CDKAL1*, *THNSL2*, *PDK2*, and *HNF1A*) were highly overlapped between datasets of TCGA LIHC, LIRI JP, and LICA FR, and *KRAS* was differentially expressed between groups (Supplemental Figure S4). After comparing *KRAS*, *Harvey Rat Sarcoma Viral Oncogene Homolog (HRAS)* and *Neuroblastoma RAS Viral Oncogene Homolog (NRAS)* levels between high‐ and low‐T cell inflammation groups, we found only *KRAS* was differentially expressed between groups. We correlated *PDK2*, *HNF1A*, and *KRAS* mRNA expression with 28 types of immune cell scores, and results showed *KRAS* was positively correlated with most immune cell types, while *PDK2* and *HNF1A* were negatively correlated with most immune cells (Figure 3).

**FIGURE 3:**
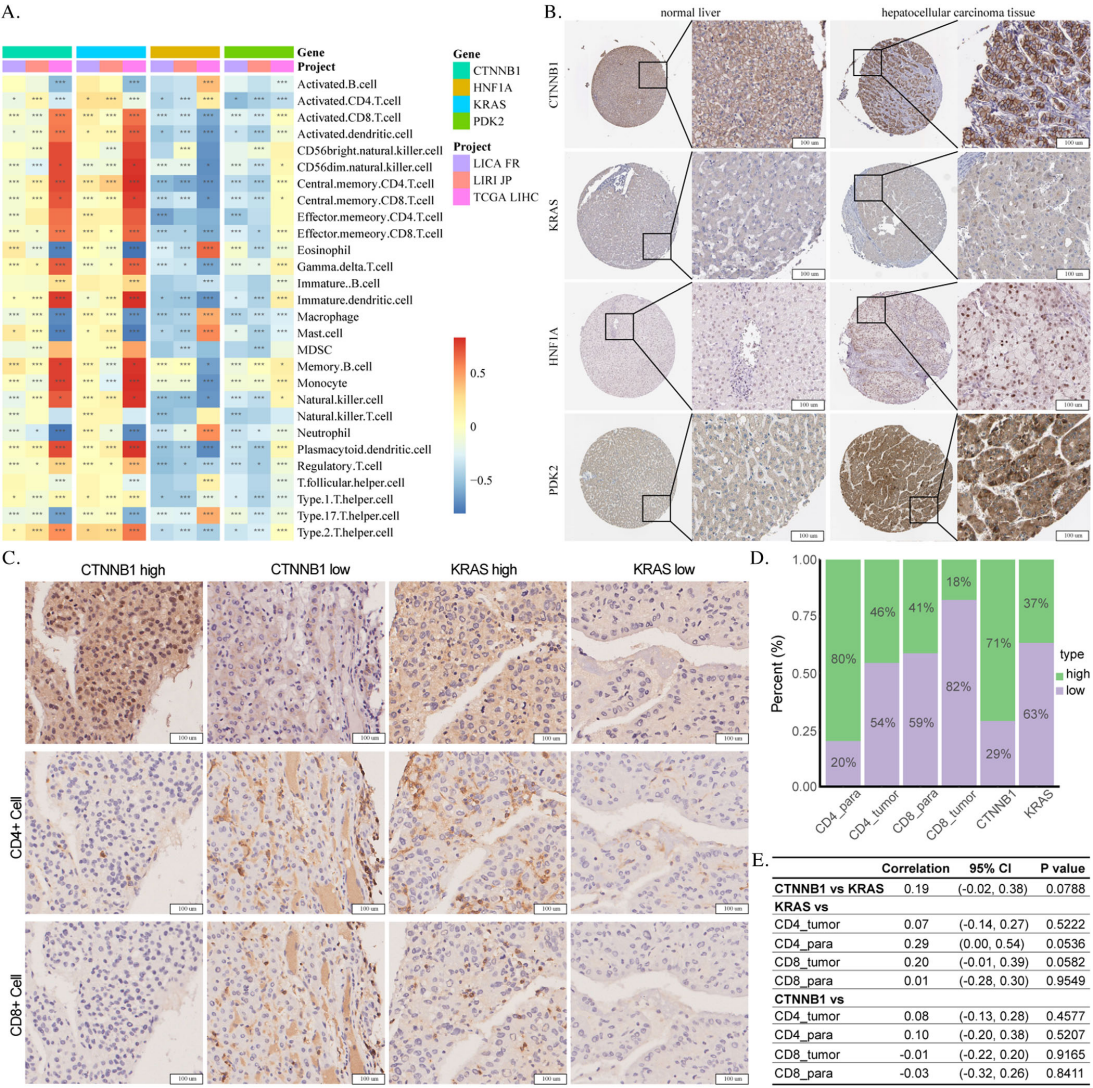
Correlation between CTNNB1, KRAS, HNF1A, PDK2, and immune cell infiltration in HCC. (A) Correlation between CTNNB1, KRAS, HNF1A, PDK2, and immune cell types in projects of LICA FR, LIRI JP, and TCGA LIHC (*p < .05, **p < .01, ***:*p *< .001). (B) Protein expression of CTNNB1, KRAS, HNF1A, and PDK2 in normal liver and HCC tissues fromHumanProteinAtlas database. (C) Protein expression of CTNNB1, KRAS, CD4, and CD8 in HCC microarray from a patient cohort of 90 patients. (D) Ratios of high‐ and low‐protein expression (CTNNB1, KRAS, CD4, and CD8) patients in microarray patient cohort. (E) Results of Spearman examination for correlation between protein expression of CTNNB1, KRAS, CD4, and CD8 in HCC tissue microarray. (CD4 and CD8 expression in tumor and paratumor area were evaluated separately.)

Former studies showed that tumor intrinsic pathways can influence immune infiltration by targeting immune regulators or chemokine axis in immune cell recruitment and differentiation.^2^, ^3^ We wondered if *KRAS*, *HNF1A*, and *PDK2* could also influence immune microenvironment of HCC through those mechanisms, and we further examined correlations between mRNA expression of *KRAS*, *HNF1A*, *PDK2*, and well‐known immune regulators, as well as chemokines. It turned out that *KRAS* was positively correlated with a series of immune stimulators and inhibitors, and across three projects, *CXCL16*, *TNFSF4*, *CD80*, *TGFBR1*, and *CCR4* were significantly correlated to *KRAS*'s expression, while *HNF1A* and *PDK2* were inversely correlated with a wide range of immune regulators and chemokines (Additional file 1–3).

Considering the limitation of bulk data yielded by sequencing technology, we corroborated results in the Human Protein Atlas database, and we found *CTNNB1* expression levels were relatively high in normal liver tissues, though carcinogenesis may additionally increase *CTNNB1* protein expression. On the contrary, protein expression of *KRAS* was not detectable in normal liver and could be found in HCC tissues. Expression of *HNF1A* and *PDK2* was increased in HCC in comparison to normal liver cells (Figure 3). In addition, we used HCC tissues microarray of 90 patients, stained by antibodies of *CD4*, *CD8*, *CTNNB1*, and *KRAS* to examine the correlation between protein expression of *CTNNB1* and *KRAS* and immune cells of CD4+ and CD8+ T cells. It demonstrated correlations between CD4+ T cells in paratumor tissues, and CD8+ T cells in tumors and *KRAS* protein expression were close to significance; high expression of *KRAS* could indicate better tumor infiltration of CD8+ T cells in HCC, though only 33 out of 90 (37%) patients demonstrated high expression of *KRAS*. Although correlations between *CTNNB1*, CD4, and CD8 protein stains were not significant, we still observed patients with high *CTNNB1* expression showed less infiltration of CD4+ and CD8+ T cells in the tissue microarray, which counted for 71% of whole patient cohort.

Former studies have shown that the ***b‐catenin*** pathway in the melanoma model could suppress recruitment of CD103+ macrophages, which play important roles in tumor‐specific CD8+ T cells’ development.^3^ Also, some studies testified *KRAS* was related to low B cell infiltration in lung adenocarcinoma, and in colorectal carcinoma, mutation of *KRAS* was related to low immune infiltration in tumor microenvironment.^4^, ^5^ The knowledge of HCC intrinsic oncogenic pathways’ influence on immune microenvironment is limited, and *CTNNB1* is deemed as a specific mutation in HCC with a high portion of patients bearing *CTTNB1* mutation classified as a worse subtype, in which patients may suffer from poor prognosis.^6^, ^7^ Our results showed that *CTNNB1* and *KRAS* were related to infiltrated CD4+ and CD8+ T cells in HCC, providing new targets for combining immune therapy and indicators for immune status evaluation. The protein expression of *KRAS* in HCC tissues was seen in only 37% of patients, while *CTNNB1* was highly expressed, which may explain the immune suppressive microenvironment in liver tumor. On the other hand, high *CTNNB1* expression could indicate high metabolic status of cancer cells, which could also depress the signals of adaptive immune responses. The explicit mechanisms concerning *KRAS*'s and *CTNNB1*'s influences on HCC immune infiltration still need further investigations to clarify.

## CONCLUSION

Tumor intrinsic pathways of *CTNNB1* and *KRAS* in HCC were related to immune infiltration levels, in which *CTNNB1* was related to low immune infiltration, while *KRAS* indicated high infiltration levels. Also, *KRAS*, *HNF1A*, and *PDK2* were highly correlated with immune regulators and the chemokine axis in HCC microenvironment, which provides new targets for combining immune therapy.

## Supporting information

Supporting Information

Supporting Information

Supporting Information

Supporting Information

Supporting Information

Supporting Information

Supporting Information

Supporting Information
