# Supplementary figures and images for: Association of intrinsic pathways with altered tumor immune infiltration in hepatocellular carcinoma: New targets for combining immune therapy
Source: Clin Transl Med. 2020 Nov 16;10(7):e219. doi: 10.1002/ctm2.219 (PMC7668290; doi:10.1002/ctm2.219)

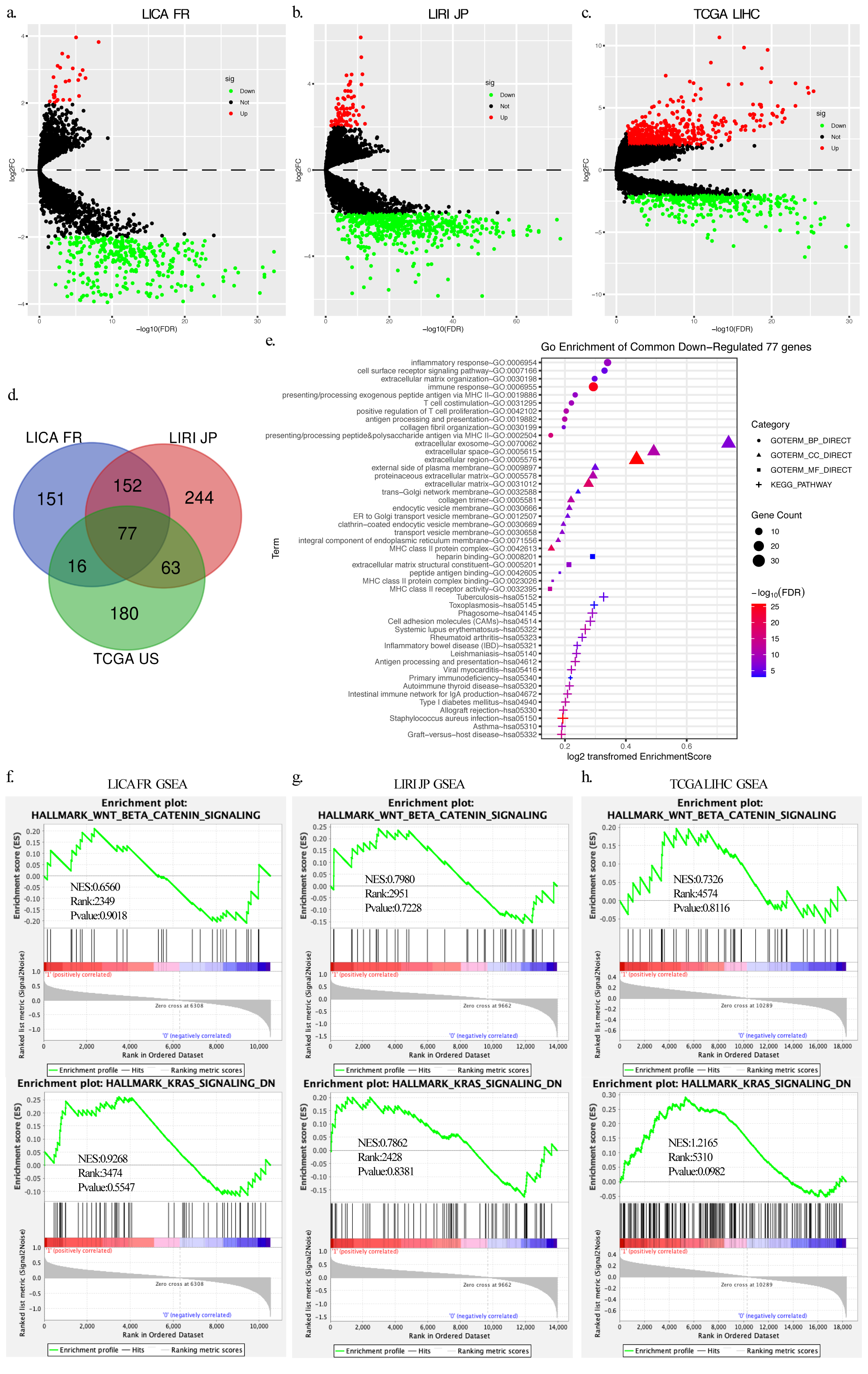

Supplement: Supplementary file 4 — Supporting Information [file CTM2-10-e219-s004.tif]

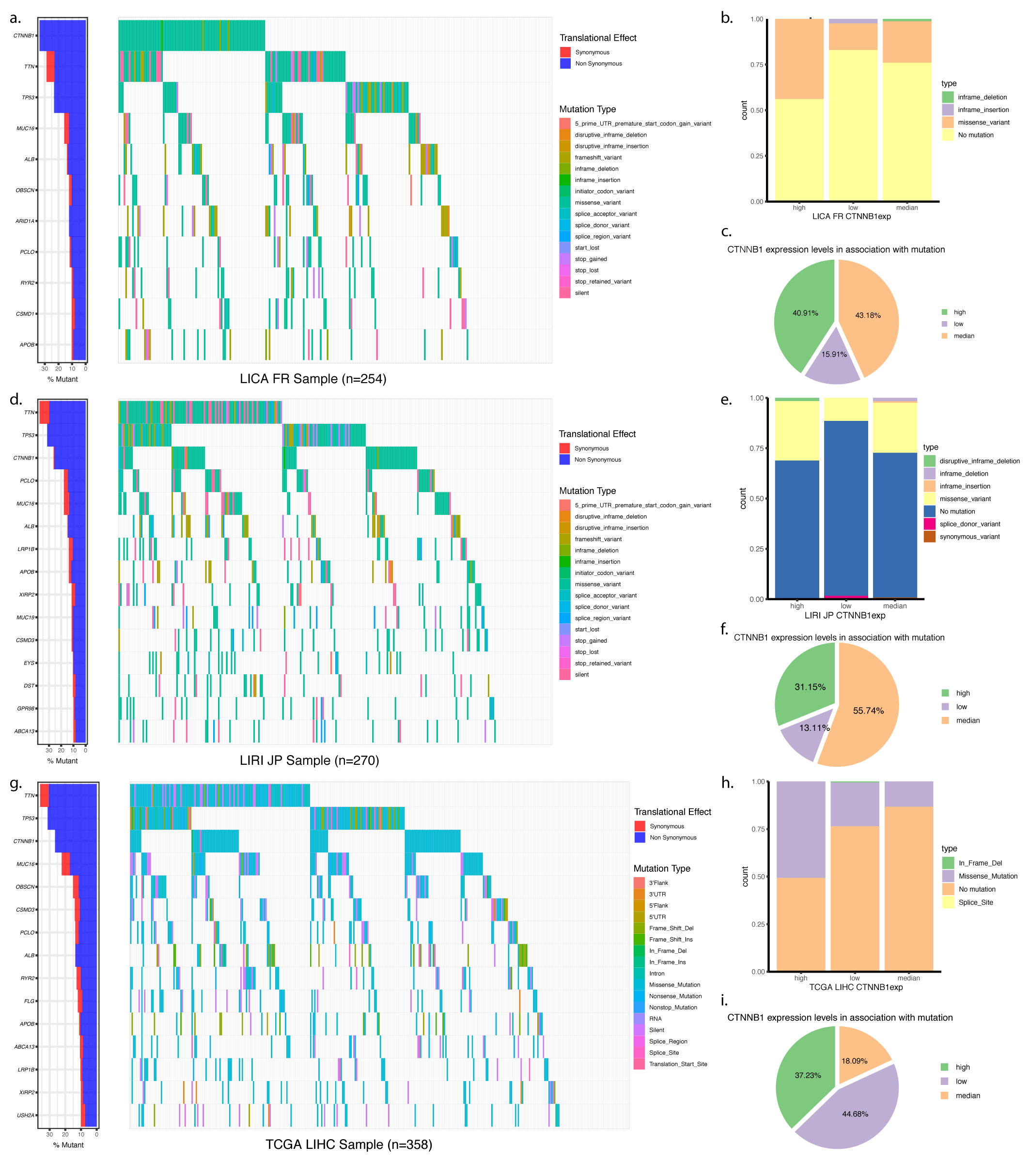

Supplement: Supplementary file 5 — Supporting Information [file CTM2-10-e219-s005.tif]

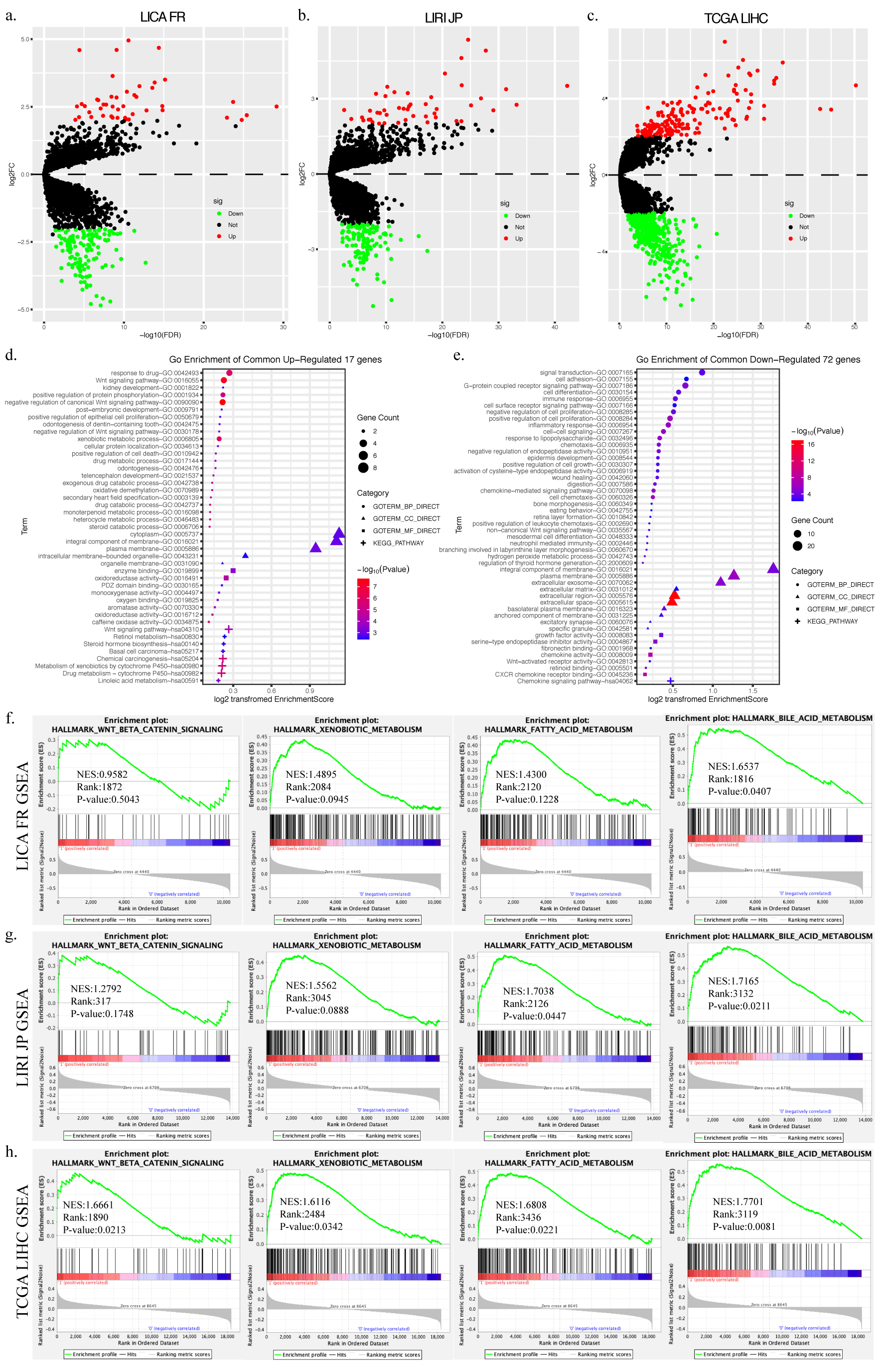

Supplement: Supplementary file 6 — Supporting Information [file CTM2-10-e219-s006.tif]

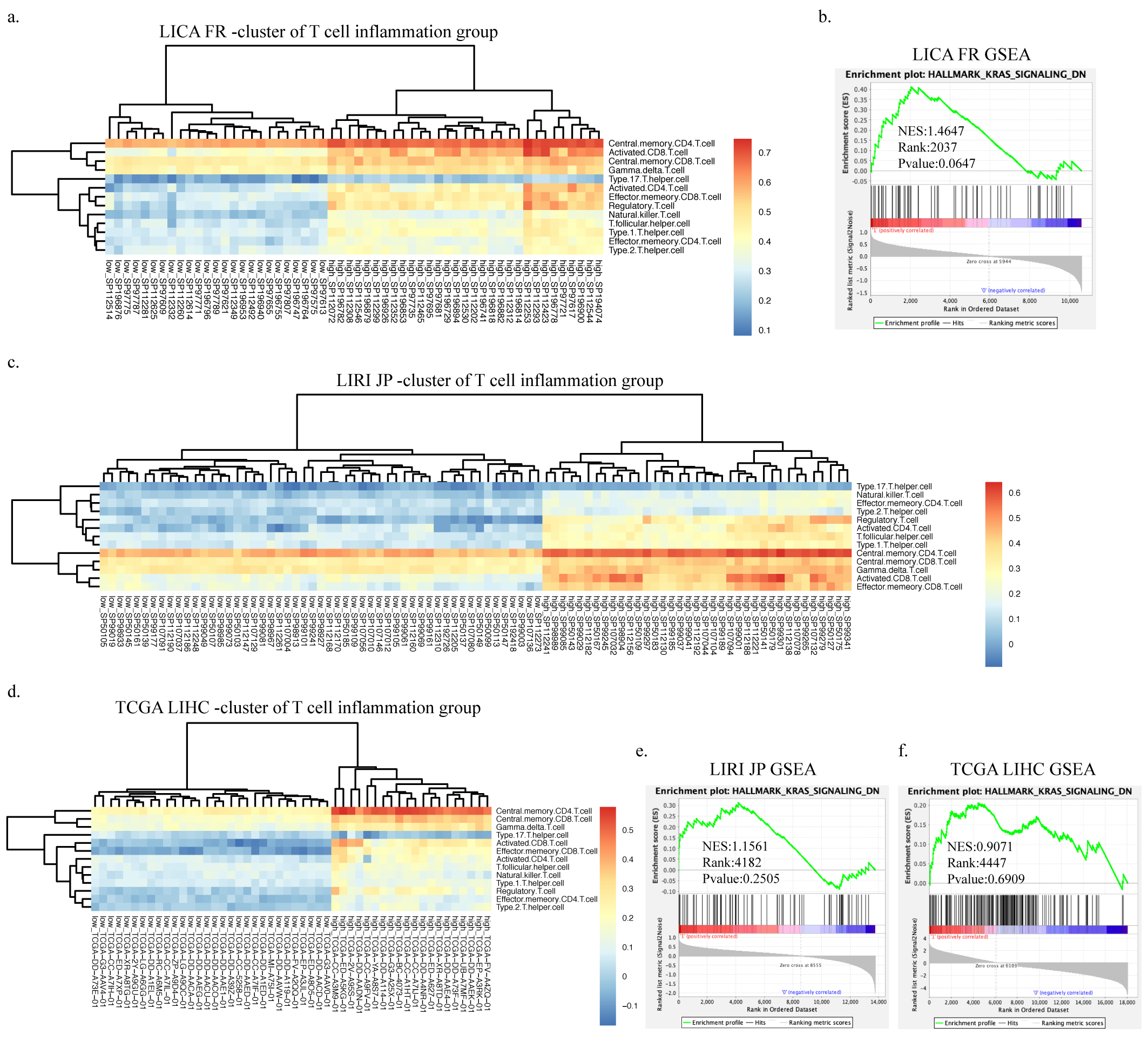

Supplement: Supplementary file 7 — Supporting Information [file CTM2-10-e219-s007.tif]

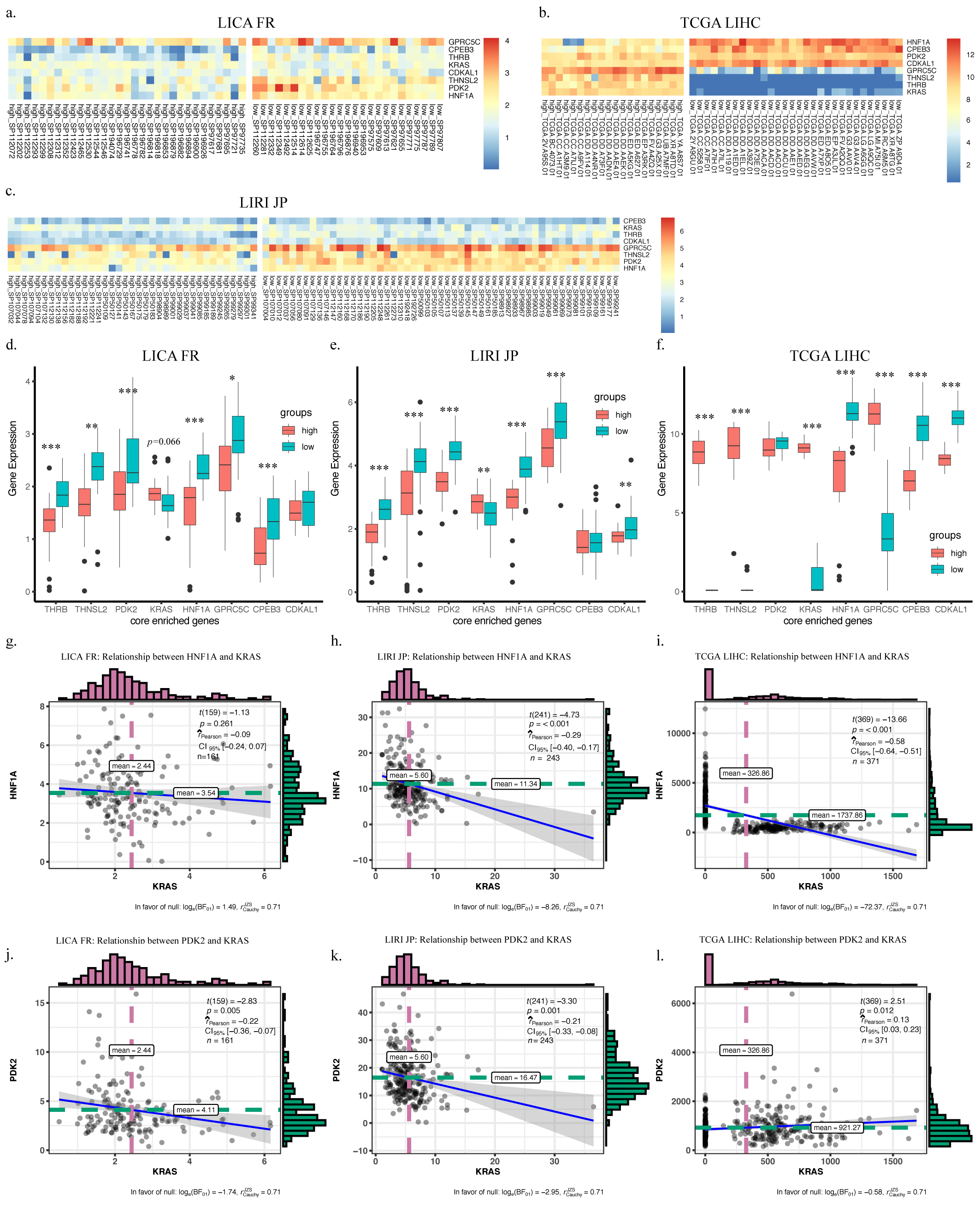

Supplement: Supplementary file 8 — Supporting Information [file CTM2-10-e219-s008.tif]
